# Assessing land use and climate-related trends in environmental quality using RSEI in a neotropical coastal basin

**DOI:** 10.1007/s10661-026-15394-y

**Published:** 2026-04-30

**Authors:** Marcelo Henrique Schmitz, José Antonio Domingues Teixeira-Junior, David Valença Dantas, Eduardo Gentil

**Affiliations:** 1 Marine Management, Ecology, and Technology Group, Department of Fishing Engineering and Biological Sciences, State University of Santa Catarina, Laguna, Santa Catarina Brazil; 2 Postgraduate Program in Territorial Planning and Socioenvironmental Development, State University of Santa Catarina, Florianópolis, Santa Catarina Brazil; 3 Postgraduate Program in Coastal and Lagoons Systems, State University of Santa Catarina, Laguna, Santa Catarina Brazil

**Keywords:** Coastal ecosystems, Decision-making, Ecological indicators, Sustainability, Water resources, Watershed

## Abstract

The Tubarão River Basin, a coastal watershed within the Atlantic Forest biome in southern Brazil, is increasingly exposed to human and climatic pressures. This study evaluated spatiotemporal patterns of terrestrial environmental quality from 2014 to 2023 using the Remote Sensing Ecological Index (RSEI), derived from Landsat-8 imagery through Principal Component Analysis (PCA). The index integrated vegetation greenness (NDVI), soil exposure (NDSI), surface moisture (LSM), and land surface temperature (LST), with NDSI and LST inverted to ensure consistent ecological interpretation and permanent water bodies masked. The first principal component (PC1) was normalized to a 0–1 scale and used as RSEI. Annual values were summarized at the municipal level, and relationships with land use were assessed using linear mixed-effects models. RSEI values remained relatively stable over time (0.69–0.80) but showed marked spatial heterogeneity. Higher values occurred in forested central and western municipalities, while lower values were associated with urbanized coastal areas. Forest cover had a significant positive effect on RSEI (*p* < 0.001), whereas agricultural and urban areas showed significant negative effects (*p* < 0.05). Despite increasing LST across municipalities, its relationship with RSEI was not significant, indicating that land-use composition outweighed isolated thermal effects. These findings highlight the utility of PCA-based RSEI for integrated environmental monitoring in tropical coastal basins.

**Supplementary Information:**

The online version contains supplementary material available at 10.1007/s10661-026-15394-y.

## Introduction

The coastal region of the Santa Catarina state (Brazil) is characterized by high biodiversity and environmental complexity, resulting from the interaction of geophysical, climatic, and ecological factors (Cristiano et al., 2018). The regional topography, strongly influenced by the Serra do Mar mountain range, regulates precipitation patterns and supports a mosaic of ecosystems such as mangroves, restingas, floodplains, coastal lagoons, and estuaries (Cristiano et al., 2018). These environments play a central role in maintaining environmental quality by regulating hydrological processes, supporting biodiversity, and sustaining ecosystem services essential to human well-being. Additionally, the interaction between the cold Malvinas Current and the warm Brazil Current forms a convergence zone that intensifies atmospheric dynamics and enhances marine productivity (Frischknecht et al., 2023). As a result, Santa Catarina represents the largest marine fishing production hub in the country (PMAP-SC, 2023).

The coastal watersheds of Santa Catarina are part of the Atlantic Forest biome, globally recognized as a biodiversity hotspot due to its high species richness and endemism (Mittermeier et al., 2004; Rezende et al., 2018; Ribeiro et al., 2009). The state still contains a significant portion of the remaining forest cover of this biome, including one of its largest continuous fragments (Ribeiro et al., 2009). These forested landscapes are fundamental for maintaining environmental quality, as they regulate surface temperature, water balance, soil stability, and ecological connectivity. Despite their ecological importance (Marques & Grelle, 2021; Rezende et al., 2018), deforestation pressures persist. In 2023 alone, approximately 12,094 hectares of Atlantic Forest were deforested nationwide, with 734 hectares occurring in Santa Catarina (Alerta Mapbiomas, 2024). Recent analyses indicate that nearly 97% of deforestation between 2019 and 2023 was driven by agricultural and livestock expansion (Alerta Mapbiomas, 2024).

Beyond deforestation, coastal watersheds in Santa Catarina are subject to multiple anthropogenic pressures, including urban expansion, population density, wastewater discharge, real estate speculation, tourism, fishing activities, and inadequate solid waste management (Barletta & Lima, 2019; Cristiano et al., 2018; Vieira et al., 2009). These pressures directly affect environmental quality by altering land cover, fragmenting habitats, and increasing surface sealing. Climate change further exacerbates these impacts through the intensification of extreme events, disruption of hydrological cycles, and rising land surface temperatures, increasing the vulnerability of coastal ecosystems (Marzouk et al., 2021; Wu et al., 2017). In response to these challenges, the State of Santa Catarina implemented the Coastal Management Plan (GERCO) in 2005, aiming to integrate environmental management, land-use planning, and coastal governance (Santa Catarina, 2005; Vieira et al., 2009). Watershed committees also play a key role in supporting participatory and integrated water resource management across extensive and ecologically sensitive areas (Couto et al., 2020; Mello et al., 2021).

In this context, the watershed emerges as a strategic spatial unit for evaluating environmental quality, as it integrates upstream and downstream processes and transcends political-administrative boundaries (Couto et al., 2020; Mello et al., 2021). Anthropogenic impacts distributed throughout the watershed directly influence water resources, landscape functioning, and coastal ecosystems (Liu et al., 2022; Wu et al., 2017). Assessing environmental quality at the watershed scale allows the identification of vulnerability patterns and supports decision-making processes aimed at sustainable territorial planning (Agrawal et al., 2022; Dahl, 2012; Lacava & Ciancia, 2020; Xu et al., 2018; Yang et al., 2022).

Remote sensing has become an essential tool for evaluating environmental quality over large spatial and temporal scales, particularly in complex and dynamic coastal regions (Guan et al., 2021; Lacava & Ciancia, 2020). By integrating indicators related to vegetation, soil exposure, moisture, and surface temperature, remote sensing enables the assessment of landscape conditions and their evolution under both anthropogenic and climatic pressures. This integrative capacity is especially relevant under current climate change scenarios, where environmental degradation may occur even in the absence of major land-use transitions (Marzouk et al., 2021; Wu et al., 2017).

Although indices such as the Remote Sensing Ecological Index (RSEI) have been widely applied to assess environmental quality in different regions (Liu et al., 2022; Wang et al., 2023; Yang et al., 2022), their application in coastal watersheds within the Atlantic Forest biome remains limited. Moreover, it is still poorly understood how environmental quality responds in contexts where land use and land cover remain relatively stable, but climatic stressors—particularly increasing land surface temperature—intensify over time. This dissociation between land-use stability and climatic pressure represents a critical knowledge gap, especially in biodiversity hotspots and densely occupied coastal basins.Based on this context, and aiming to advance the understanding of environmental quality dynamics in coastal watersheds, this study investigates the Tubarão River Basin using an integrated remote sensing approach. Specifically, the objectives were to: (i) analyze spatiotemporal patterns of terrestrial environmental quality in the basin between 2014 and 2023 using the RSEI; (ii) assess the relationship between terrestrial environmental quality and land use and cover composition; and (iii) contextualize observed changes in environmental quality in light of regional climatic tendencies and urban expansion.

## Material and methods

### Study area

**Fig. 1:**
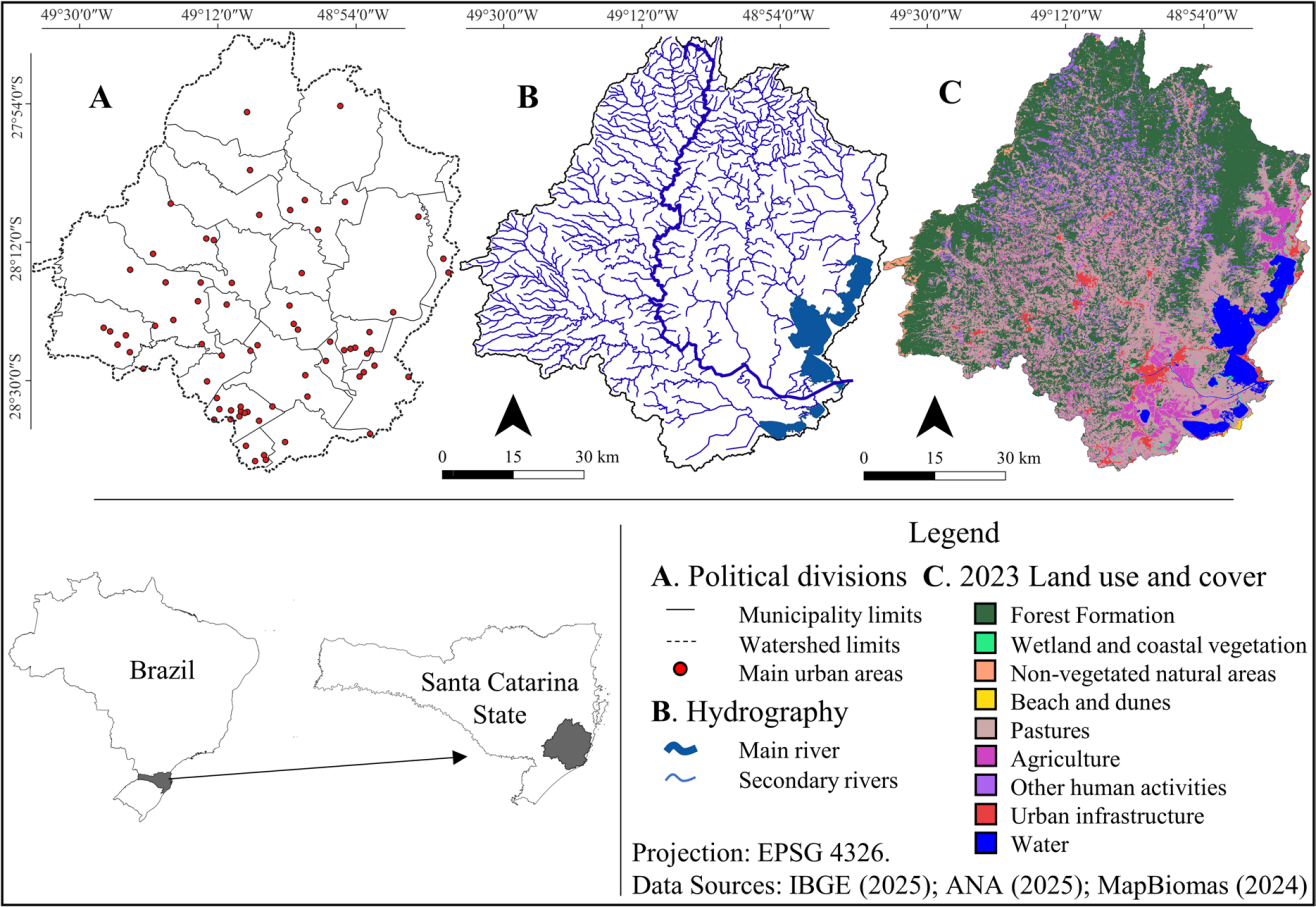
Map of the Tubarão River Basin, Lagoon Complex, and Adjacent Basins. **A** Division of municipalities and locations within the basin (IBGE, 2025); **B** Hydrography (ANA, 2025); **C** 2023 Land use and land cover (Mapbiomas, 2024)

The Tubarão River Basin drainage area spans approximately 5,900 km^2^ in the coastal region of the state of Santa Catarina (Fig. 1). It is part of the “State Coastal Basins of SC” Water Resource Management Unit. A total of 25 municipalities are located partially or entirely within the Tubarão River Basin drainage area, with its waterways serving multiple uses, providing both ecological and socioeconomic services. According to 2023 data from the MapBiomas project, about 44% of the basin’s landscape was covered by forest formation, and 36% by pastures. In the same year, agriculture and urban infrastructure represented 5% and 2%, respectively (Mapbiomas, 2024). Spatializing these data (Fig. 1C) reveals a high rate of fragmentation of natural vegetation. Except for the large vegetated areas in the north and west, primarily related to the Santa Catarina mountain ranges, the remaining Atlantic Forest fragments are small and scattered. The coastal region of the basin, on the other hand, is predominantly composed of urban infrastructure and extensive areas of pasture and agriculture.

The basin is predominantly influenced by a humid subtropical climate (Cfa, Köppen–Geiger), with mean annual precipitation ranging from approximately 1,400 to 1,800 mm and mean temperatures between 14 and 24 °C (Águas de Santa Catarina, 2001). Vegetation is mainly composed of Atlantic Forest formations, including dense ombrophilous forests in mountainous areas, coastal vegetation complexes, and riparian and floodplain forests along the drainage network.

The socio-environmental relevance of this basin lies in its high diversity of ecosystems, including rivers, streams, mangroves, estuaries, coastal dune systems, and remnants of the Atlantic Forest. The Santo Antônio dos Anjos/Imaruí lagoon complex, the final destination of the Tubarão River Basin drainage, also plays a crucial role in regulating water quality by acting as a natural filter that removes sediments and pollutants before they are discharged into the ocean. Furthermore, this ecosystem serves as a vital nursery and feeding area for birds, fish, and invertebrates, supporting the maintenance of populations of species important to artisanal, recreational, and industrial fishing (Barletta et al., 2019; Frischknecht et al., 2023; Pimenta et al., 2024).

### Remote sensing ecological index

In this study, the Remote Sensing Ecological Index (RSEI) was applied to assess terrestrial environmental quality using land-surface indicators derived from orbital remote sensing data. The RSEI has been widely employed to evaluate environmental conditions across large geographical areas (Boori et al., 2021; Liu et al., 2022; Wang et al., 2023; Xu et al., 2018; Yang et al., 2022). The RSEI is composed of four indicators: Normalized Difference Vegetation Index (NDVI), Normalized Difference Soil Index (NDSI), Land Surface Moisture (LSM), and Land Surface Temperature (LST). These four indicators reflect the landscape’s attributes of vegetation, aridity, moisture, and heat (greenness, dryness, wetness, and heat) (Wang et al., 2023; Xu et al., 2018). The application of the RSEI represents an approach following the pressure-state-response framework recommended by the Organization for Economic Co-operation and Development (OECD, 2003). In this model, NDSI represented the intensity of anthropogenic pressure on the environment, NDVI represented the environmental state, and LSM and LST indicators reflected the landscape’s climatic response to environmental changes (Boori et al., 2021; OECD, 2003; Wang et al., 2023).

The RSEI was calculated annually for the Tubarão River Basin over the period from 2014 to 2023. LandSat-8 Collection 2 products corresponding to the study area (path/row 220/79 and 220/80) were used. The search for LandSat products was conducted within a three-month temporal window, between August and October of the evaluated years. This period was selected due to the higher rainfall in the Tubarão River Basin, which directly affects the NDVI, moisture (LSM), and temperature (LST) indicators. Permanent water bodies were masked prior to the calculation of all sub-indexes to prevent their influence on RSEI values.

The NDVI and LSM indicators were calculated using Eqs. 1 and 2, as described by Wang et al. (2023). The LST indicator was calculated according to Eq. 3, using values derived from Eqs. 4, 5, and 6 (Estoque et al., 2017) and emissivity values reported in Sobrino et al. (2004). The NDSI indicator was calculated using Eq. 7 (Xu et al., 2018). The use of the NDSI indicator is highly recommended, as it allows for the identification of both impervious surfaces and soil features, without the need for water surface removal during the preprocessing stage of the scenes (Xu et al., 2018).

Equation 1. Normalized Difference Vegetation Index – NDVI (Wang et al., 2023). In which ρ denotes the reflectance in the respective bands.

1 $$NDVI=\frac{\rho NIR-\rho RED}{\rho NIR+\rho RED}$$

Equation 2. Land Surface Moisture – LSM (Wang et al., 2023). In which ρ denotes the reflectance in the respective bands.

2 $$LSM=\mathrm{0,1511}\rho BLUE+\mathrm{0,1972}\rho GREEN+\mathrm{0,3283}\rho RED+\mathrm{0,3407}\rho NIR-\mathrm{0,7117}\rho SWIR1-\mathrm{0,4559}\rho SWIR2$$

Equations 3, 4, 5, and 6. Land Surface Temperature – LST (Estoque et al., 2017). In which T_B_ is the LandSat 8 band 10 brightness temperature; λ = Wavelength of the emitted radiance (λ = center wavelength of band 10 of Landsat 8 = 10.8 μm); ρ is calculated using the Boltzmann constant (σ = 1.38 × 10⁻^23^ J/K), Planck’s constant (h = 6.626 × 10⁻^34^ Js), and the speed of light (c = 2.998 × 10⁸ m/s) (Eq. 4); ε = land surface emissivity (Eq. 5), where m = 0.004 and *n* = 0.986 are reported in Sobrino et al. (2004) and Pv is given by Eq. 6.

3 $$LST\left(^\circ C\right)=\frac{{T}_{B}}{1+\left(\lambda x\frac{{T}_{B}}{\rho }\right)lnln\varepsilon }$$

4 $$\rho =h\frac{c}{\sigma }=\mathrm{1,438}x{10}^{-2}mK$$

5 $$\varepsilon =m{P}_{v}+n$$

6 $${P}_{v}={\left(\frac{NDVI-{NDVI}_{min}}{{NDVI}_{max}-{NDVI}_{min}}\right)}^{2}$$

Equation 7. Normalized Difference Soil Index – NDSI (Xu et al., 2018). In which ρ denotes the reflectance in the respective bands.

7 $$NDSI=\frac{\rho MIR1-\rho NIR}{\rho MIR1+\rho NIR}$$

The spectral bands used in the computation of NDVI, LSM, and NDSI followed the Landsat-8 OLI configuration, with BLUE (Band 2), GREEN (Band 3), RED (Band 4), NIR (Band 5), SWIR1 (Band 6), and SWIR2 (Band 7).

To ensure a consistent ecological interpretation, land surface temperature (LST) and the Normalized Difference Soil Index (NDSI) were inverted prior to the Principal Component Analysis so that higher values in all input indicators represented better environmental conditions. As a result, the first principal component (PC1), which concentrated the shared variance among vegetation, landscape structure, surface moisture, and thermal conditions, consistently reflected an integrated gradient of environmental quality and was therefore adopted as the Remote Sensing Ecological Index (RSEI). The PC1 scores were subsequently normalized to a 0–1 range to enable spatial comparison and temporal consistency across the analyzed period.

### Statistical analysis

After computing the RSEI for all years, an annual mean RSEI value was determined for each municipality by averaging the values of all its pixels. Subsequently, based on the annual mean RSEI values, the Euclidean distance between municipalities was calculated to assess their similarity in environmental conditions. Finally, using the Euclidean distance, these municipalities were grouped into clusters using the “hclust” function in R, allowing for the identification of distinct patterns and trends across the study area.

In order to examine the possible effects of land use and cover composition over environmental quality, a Linear Mixed Model (LMM) was used to examine the effects of the land use categories "Forest", "Agriculture", and "Urban Area" on the RSEI. These variables were treated as fixed effects, assessing their direct impact on environmental quality. The "Forest" variable aimed to evaluate how forest cover influences environmental quality, while "Agriculture" was included to explore its potential negative effects on the RSEI. The "Urban Area" variable was examined to understand the impact of urbanization, with the expectation that it would reduce the RSEI. A random effect for the "Municipality" variable was also included to account for municipality-specific factors influencing environmental quality. The analysis was performed using R software, and statistical significance was assessed through the Chi-square test.

Additionally, a post hoc mixed-effects analysis was performed to evaluate the relationship between annual land surface temperature (LST) and the PCA-derived RSEI. Municipality-level annual mean values were used, with municipality included as a random intercept to account for repeated measurements and spatial heterogeneity. This analysis was conducted to complement the PCA results and to assess the potential influence of regional warming on integrated environmental quality.

**Fig. 2:**
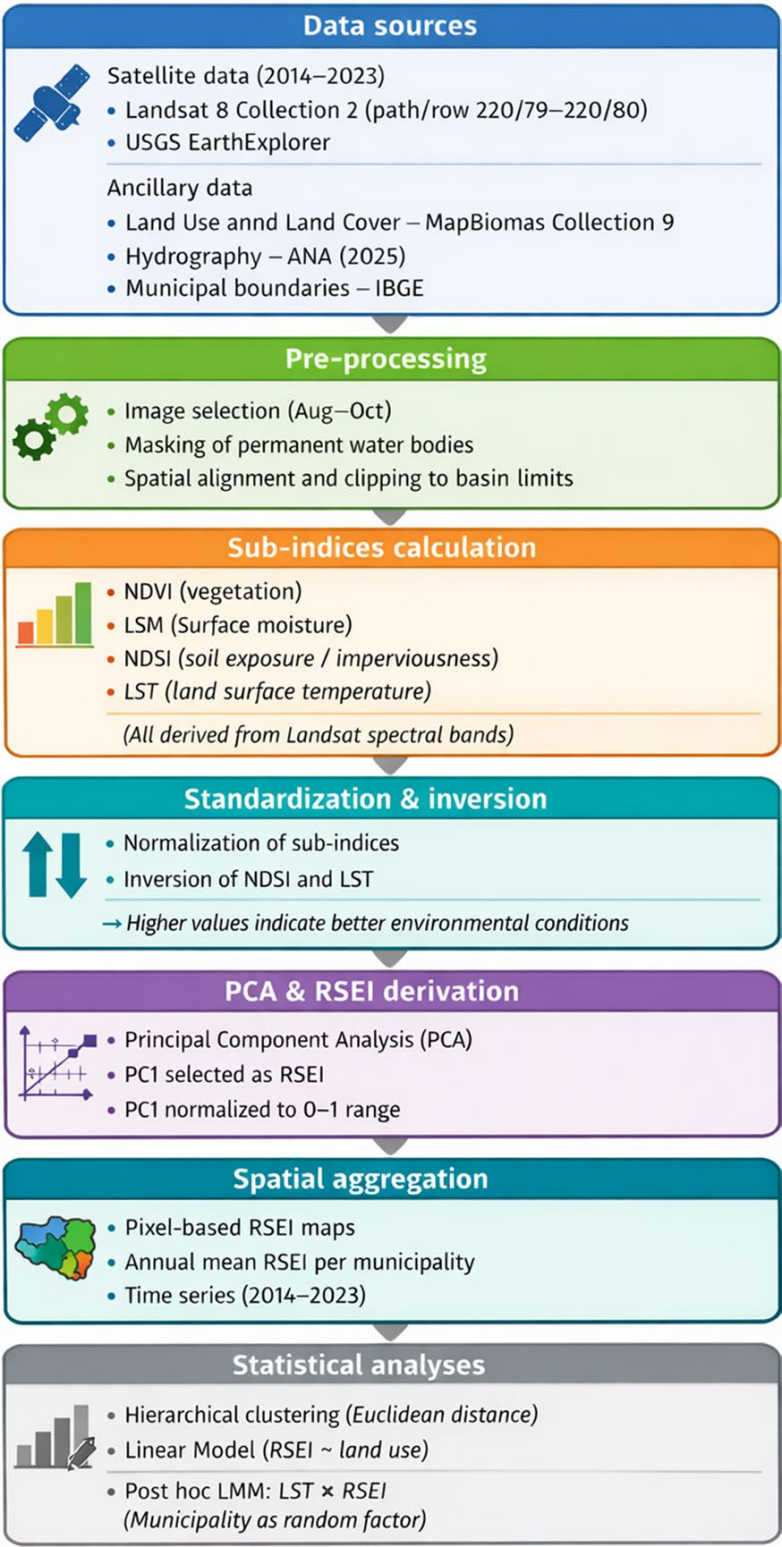
Schematic overview of the methodological workflow adopted in this study, including data acquisition, remote sensing processing, RSEI construction through PCA, spatial aggregation at the municipal level, clustering analysis, and mixed-effects statistical modeling

All indices were generated and spatially processed in QGIS, while all statistical analyses were performed in R. Satellite images were obtained from the USGS EarthExplorer platform (https://earthexplorer.usgs.gov), land use and land cover data were downloaded from MapBiomas Collection 9 (MapBiomas, 2024), and hydrographic data were obtained from the Brazilian National Water Agency (ANA, 2025). Figure 2 presents a schematic summary of the methodological workflow adopted in this study.

## Results

**Fig. 3:**
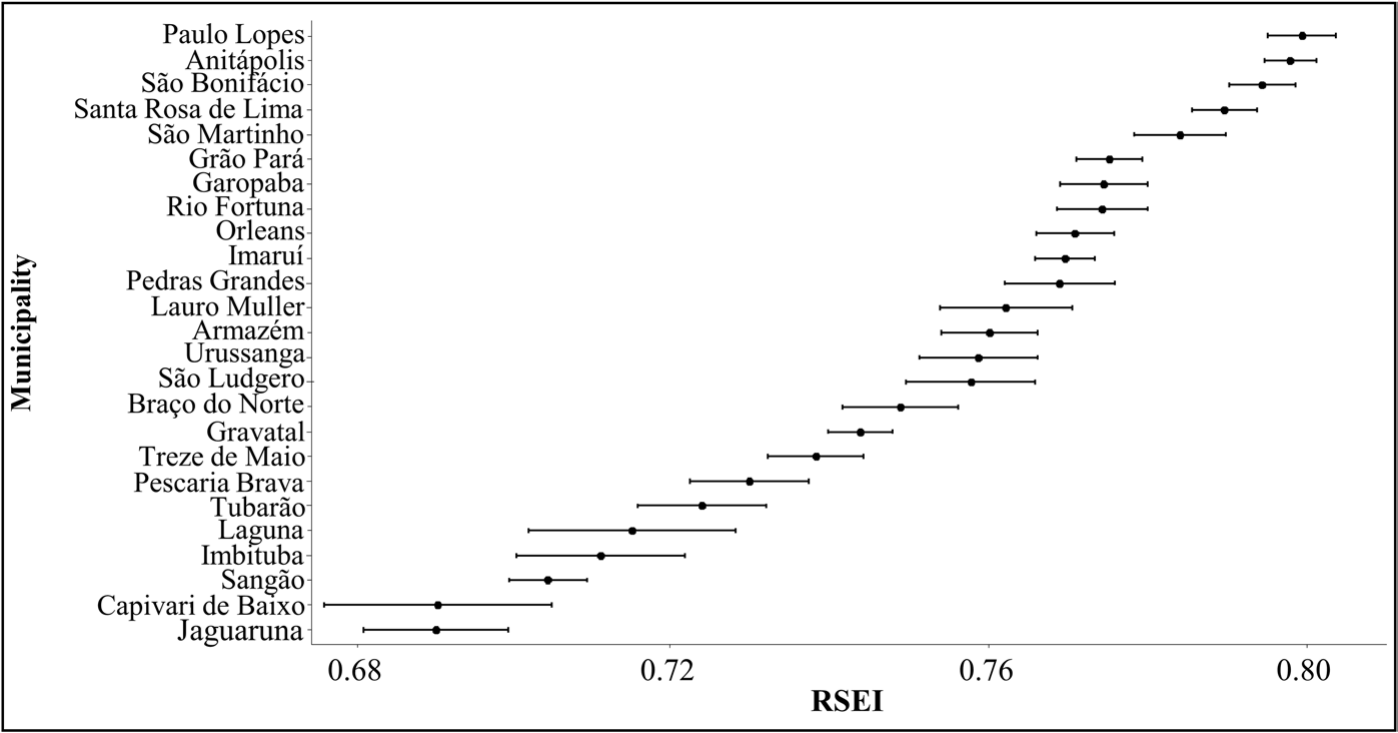
Mean Remote Sensing Ecological Index (RSEI) values (± standard deviation) for municipalities in the Tubarão River Basin from 2014 to 2023

**Fig. 4:**
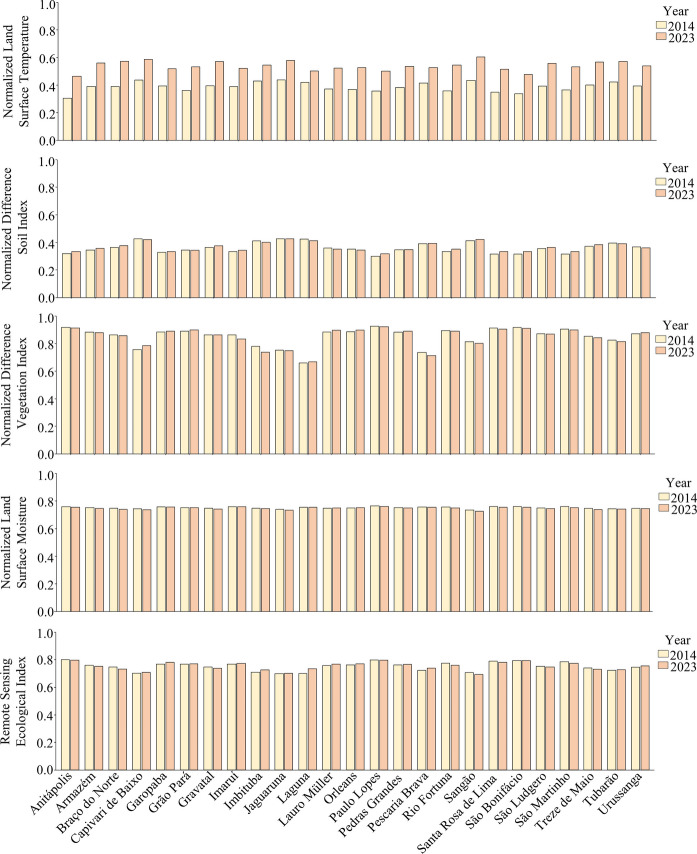
Values the Remote Sensing Ecological Index and its components for each municipality in 2014 and 2023

Overall, the RSEI values, which range from 0 to 1, did not exhibit extreme variations in the Tubarão River Basin, fluctuating between 0.69 ± 0.01 and 0.80 ± 0.01 (Fig. 3). Among the municipalities, Paulo Lopes had the highest mean RSEI from 2014 to 2023, while Jaguaruna recorded the lowest. Regarding the RSEI components, the mean municipal LSM, NDVI, and NDSI exhibited smooth variations between 2014 and 2023, while the LST increased in all municipalities (Fig. 4) and in the whole basin (Supplementary Material 1). The loadings and the proportion of variance explained by the principal components used to derive the RSEI are provided in Supplementary Material 1. Across the study period, the first principal component explained, on average, 67.0 ± 2.4% of the total variance, indicating that it provides a suitable integrative basis for RSEI derivation.

**Fig. 5:**
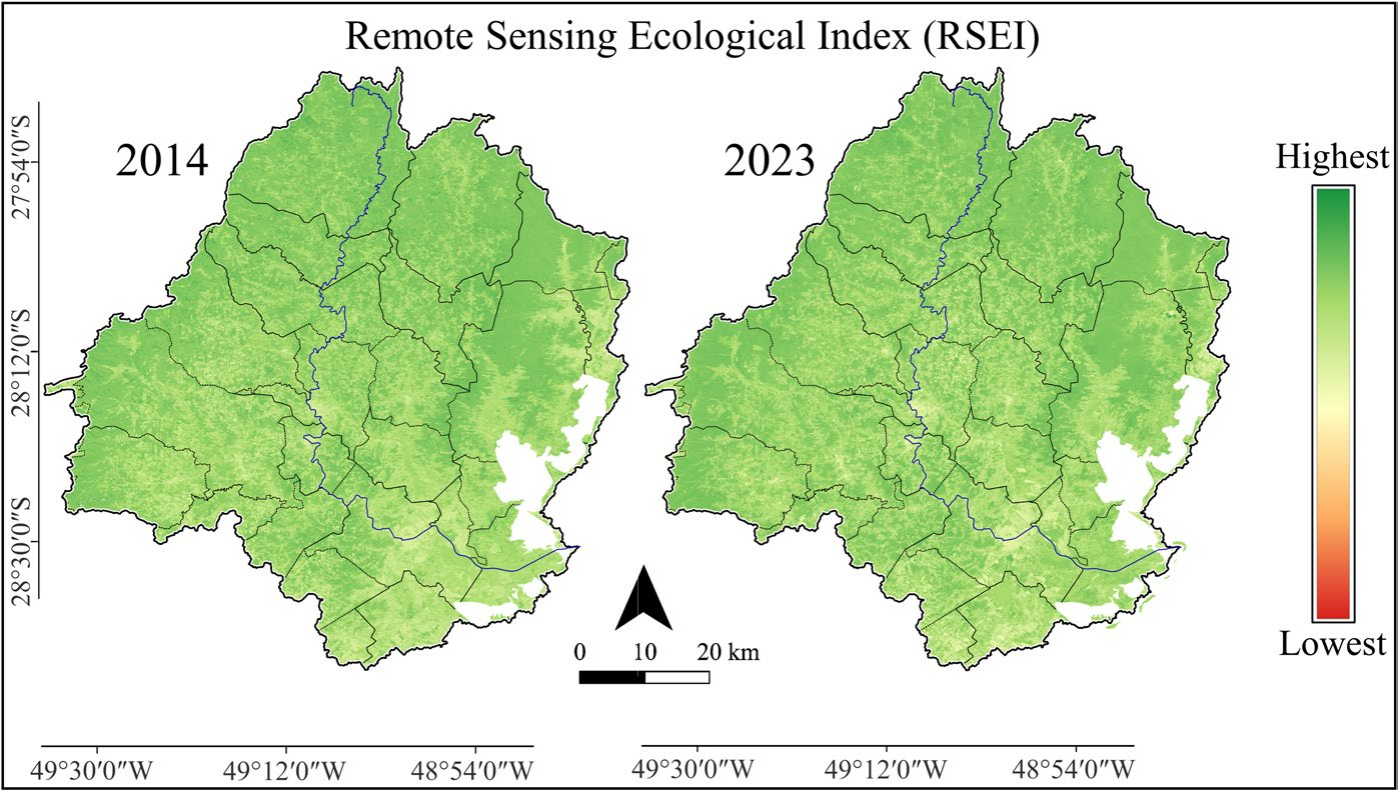
Maps of 2014 (left) and 2023 (right) for the Remote Sensing Ecological Index (RSEI) in the Tubarão River Basin

The visual analysis of the RSEI patterns indicates that the spatial distribution of RSEI in the Tubarão River Basin remained relatively consistent from 2014 to 2023 (Fig. 5).

**Fig. 6:**
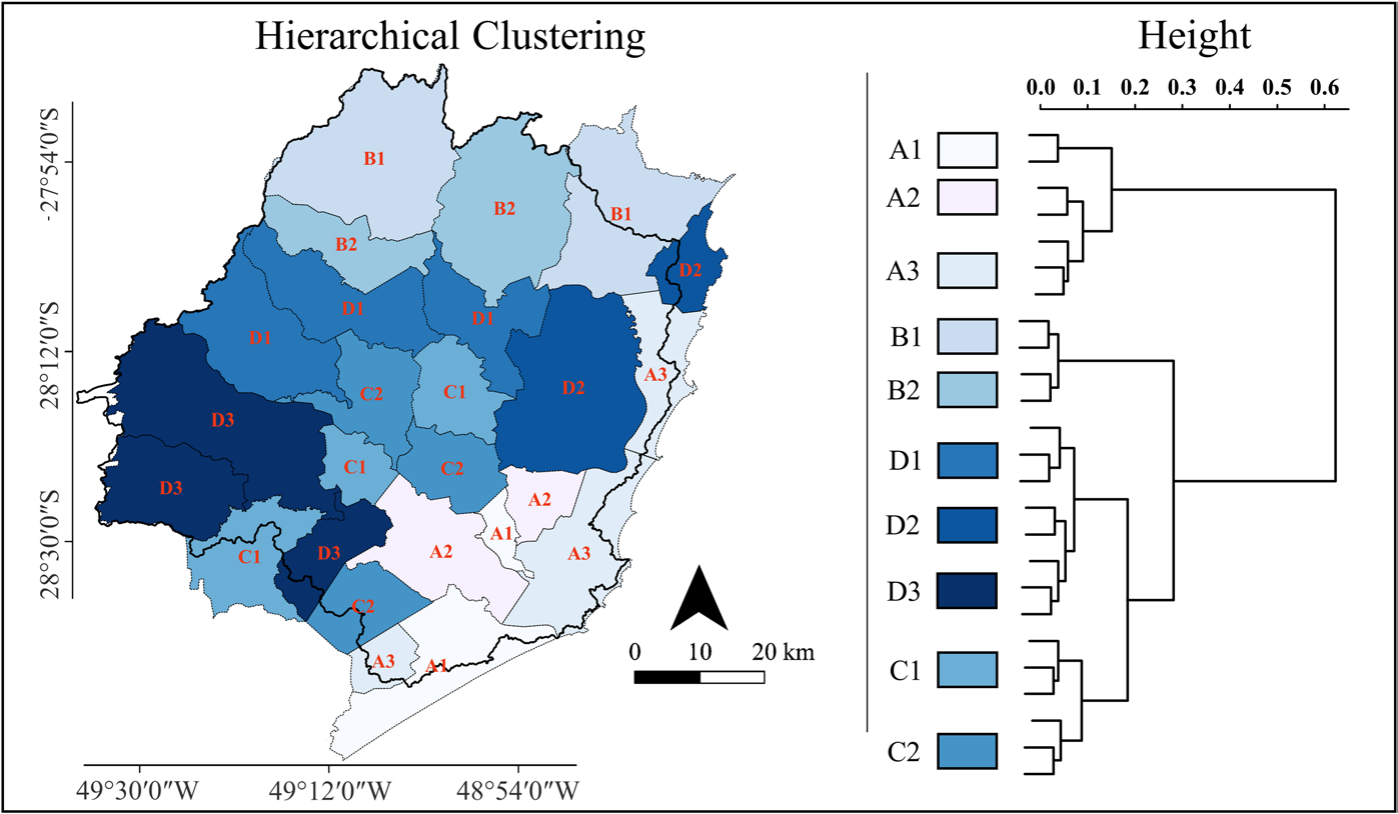
Hierarchical clustering of the Remote Sensing Ecological Index (RSEI) based on Euclidean distances between municipal mean RSEI values (right), and spatial distribution of clusters (left), with darker shades indicating higher RSEI values. The solid black line represents the Tubarão River Basin limits

The spatialization of the hierarchical clustering revealed a distinct spatial pattern in environmental quality across the region (Fig. 6). The central and western municipalities exhibited higher RSEI values, as indicated by the darker blue shades representing clusters C and D. In contrast, the southeastern and coastal municipalities showed lower RSEI values, corresponding to clusters A1, A2, and A3 which are represented by lighter shades. The dendrogram further supports this classification, illustrating a clear hierarchical structure where municipalities with similar environmental quality are grouped together.

**Fig. 7:**
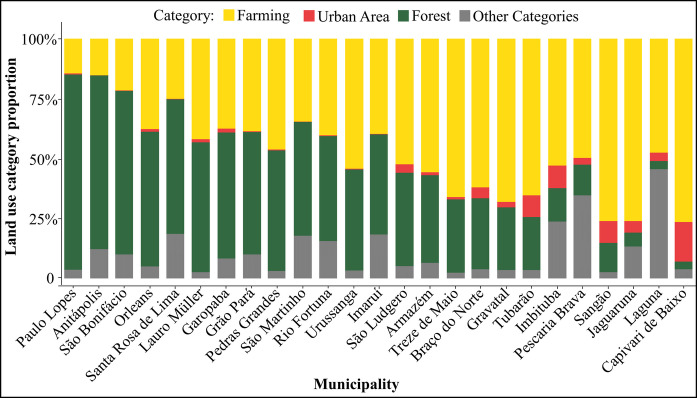
Mean land use and land cover proportions for municipalities in the Tubarão River Basin (2014–2023). Values represent the average annual proportion of each category per municipality; remaining area required to complete 100% is grouped as Other Categories. Colors indicate Farming (yellow), Urban Area (red), Forest (green), and Other Categories (gray). Municipalities are ordered from highest to lowest forest proportion

The land use distribution in the Tubarão River Basin municipalities was predominantly composed of forest and farming areas, which together accounted for the largest proportions in most municipalities (Fig. 7). Paulo Lopes exhibited the highest forest coverage, while Capivari de Baixo had the lowest. The municipalities with the highest forest proportions tended to have the lowest farming proportions. The order in which the municipalities appear in the figure, from those with higher forest proportions to those with lower ones, was similar to the ranking of municipalities with higher RSEI values presented in Fig. 3, suggesting a visual similarity between forest coverage and environmental quality.

The mixed-effects model revealed a strong positive effect of forest cover on RSEI (t = 6.08), indicating a positive association between forest cover and environmental quality. In contrast, agricultural and urban land uses exhibited significant negative effects, with urban areas showing the strongest association with RSEI reduction (t = −2.74). Approximately 55% of the total variance was explained by municipality-level differences, highlighting the importance of spatial heterogeneity in shaping environmental quality patterns (Table 1).

**Table 1:** Results of the Linear Mixed Model (LMM) for the RSEI index considering the fixed effects of land use categories and the random effect of municipality. * Indicates statistically significant values

Variable	Estimate	Std. Error	*t* value	Chi-square	*p*-value
Intercept	0.742	0.014	54.593	2980.380	< 2.2 e^−16^ *
Forest	0.094	0.015	6.075	19.152	1.2 e^−9^ *
Farming	−0.040	0.018	−2.302	5.301	0.021 *
Urban Area	−0.157	0.057	−2.739	7.502	0.006 *

The mixed-effects model indicated a negative but non-significant association between annual LST and RSEI (estimate =  − 0.006, t =  − 1.45), suggesting that interannual temperature variability alone does not strongly explain changes in environmental quality at the municipal scale. Most of the variance in RSEI was attributed to municipality-level differences, indicating that spatial heterogeneity outweighs temporal thermal effects.

## Discussion

This study elucidates the relationship between land use composition and terrestrial environmental quality in the Tubarão River Basin, a landscape dominated by forested and agricultural areas. RSEI values remained relatively stable between 2014 and 2023, varying within a narrow range from 0.69 ± 0.01 to 0.80 ± 0.01 across municipalities. Higher RSEI values were consistently associated with forested municipalities in the central and western portions of the basin, whereas lower values characterized the more urbanized coastal areas, a spatial pattern corroborated by hierarchical clustering. Mixed-effects modelling indicated a positive and significant influence of forest cover on environmental quality, while agricultural and urban areas exerted significant negative effects. In contrast to the relative stability of land use and land cover proportions over the study period, land surface temperature increased in all municipalities. However, a post hoc mixed-effects analysis indicated only a negative trend between annual LST and the PCA-derived RSEI (i.e., not statistically significant), suggesting that interannual temperature variability alone did not strongly explain differences in integrated environmental quality at the municipal scale. Overall, the results indicate that spatial heterogeneity and land-use composition outweighed the isolated effect of LST in shaping RSEI patterns. These patterns reflect the combined behavior of vegetation greenness (NDVI), surface moisture (LSM), soil exposure (NDSI), and thermal conditions (LST), highlighting the integrative nature of the PCA-derived RSEI.

Santa Catarina state lies entirely within the Atlantic Forest biome, which is globally recognized as a biodiversity hotspot (Marques & Grelle, 2021; Mittermeier et al., 2004; Rezende et al., 2018; Ribeiro et al., 2009). This biome plays a crucial role in carbon sequestration, biodiversity conservation, climate regulation, and water cycle maintenance (Marques & Grelle, 2021; Rezende et al., 2018), supporting both local and national ecological balance. Thereby, the presence of vegetation cover is expected to directly influence the RSEI key components. The presence of vegetation contributes to lower land surface temperature through shading and evapotranspiration (Estoque et al., 2017). Additionally, forests maintain higher vegetation indices by sustaining dense and healthy vegetation (Guan et al., 2021), enhance land surface moisture by improving water retention, and reduce soil exposure, preventing land degradation. Together, these processes explain why forested landscapes consistently exhibited higher RSEI values, reinforcing the role of vegetation structure as a key determinant of terrestrial environmental quality.

The Tubarão River Basin, although entirely within the Atlantic Forest biome, features diverse vegetation types shaped by topography and proximity to the coast. Different land use categories exhibit varying responses in RSEI values (Zhu et al., 2021), with forests generally showing higher values compared to shrubby vegetation (Guan et al., 2021; Zhu et al., 2021). The Serra region, located along the western boundary of the basin, is dominated by dense montane and submontane forests, which exhibit higher vegetation indices. In contrast, coastal areas, characterized by restinga and mangrove vegetation, tend to show lower vegetation index values, despite playing important ecological roles. Combined with the intense urbanization along the coast (Cristiano et al., 2018; Vieira et al., 2009), this pattern likely influenced the spatial distribution of RSEI values across the basin, with forested regions contributing more effectively to environmental quality, while urbanized coastal zones—despite their vegetation—showing lower index values. This pattern is consistent with findings in other studies using RSEI, such as Liao et al. (2020), Liu et al. (2022), and Yang et al. (2022), which also highlight the relationship between land cover types and environmental quality.

Urbanization is widely recognized as a major driver of environmental degradation (Wang et al., 2023; Xu et al., 2018). The conversion of natural land cover into impervious surfaces increases surface runoff, reduces vegetation cover, and elevates land surface temperatures due to the urban heat island effect, all of which contribute to environmental decline (Estoque et al., 2017; Wang et al., 2023; Xu et al., 2018). In the Tubarão River Basin, rapid urban expansion in recent decades, particularly in coastal municipalities, has intensified environmental pressures driven by tourism-related development and land use changes (Cristiano et al., 2018; Vieira et al., 2009). Consequently, these areas have exhibited lower RSEI values, indicating reduced environmental quality. The expansion of impervious surfaces, closely linked to increasing population density, may be a key factor in this degradation. Xu et al. (2018) highlighted that these surfaces contribute to declining RSEI values by increasing runoff, reducing water infiltration, and raising mean land surface temperatures. As a result, areas experiencing dense urbanization and infrastructure expansion are particularly affected.

Although agriculture exhibited a statistically significant negative effect on RSEI, its influence on environmental quality is complex and strongly dependent on crop type and management practices (Escobar et al., 2020; Strassburg et al., 2014). In the Tubarão River Basin, a substantial proportion of agricultural land—approximately 23%—is occupied by irrigated rice cultivation (MapBiomas, 2024), which differs markedly from other agricultural systems in terms of ecological functioning (Manjula et al., 2023). Flooded rice paddies tend to increase NDVI and land surface moisture while simultaneously reducing soil exposure and land surface temperature, partially offsetting the negative signals typically associated with agricultural land use (Manjula et al., 2023). As a result, although agriculture exerted an overall negative influence on integrated environmental quality, the predominance of irrigated rice fields likely moderated the magnitude of this effect, distinguishing it from the impacts of other agricultural practices such as pasture expansion and annual dryland crops.

The RSEI values remained relatively stable across municipalities in the Tubarão River Basin between 2014 and 2023, without evidence of a consistent temporal decline. During the same period, forest cover showed only minor variation (from 44.9% of the basin in 2014 to 44.4% in 2023), and urban areas exhibited limited expansion (from 0.08% to 0.12%). This overall land-use stability indicates that temporal fluctuations in RSEI were not primarily driven by changes in land cover composition. While land use patterns help explain the spatial distribution of environmental quality across the basin (Zhu et al., 2021), land use dynamics alone do not account for the observed interannual variability in RSEI values. Instead, these results suggest that additional environmental drivers, such as regional climatic variability, may modulate short-term changes in integrated environmental quality.

One possible contributing factor is the influence of climate change, as rising temperatures—evidenced by the increase in LST across all municipalities during the study period—may alter precipitation regimes and affect key land-surface processes related to environmental quality (Marzouk et al., 2021; Wang et al., 2023; Wu et al., 2017; Yang et al., 2022). Higher temperatures can enhance evapotranspiration rates and influence surface moisture dynamics, while more frequent extreme weather events may exacerbate environmental stress, particularly in vulnerable coastal landscapes (Barletta & Lima, 2019; Cristiano et al., 2018; Marzouk et al., 2021; Wu et al., 2017). However, the post hoc analysis indicated that LST alone was not a significant predictor of RSEI, suggesting that temperature increases may act as a background stressor rather than a dominant driver of environmental quality. Therefore, even in the absence of pronounced land-use changes, broader climatic variability may contribute to interannual fluctuations, although this effect could not be isolated from other interacting drivers in the present analysis. Our objective was not to reconstruct long-term historical trajectories, but to assess recent patterns of environmental quality and warming that are most relevant to current management and territorial planning contexts. Accordingly, the implications of this temporal window are discussed in terms of short- to medium-term environmental dynamics rather than long-term climatic trends. This temporal scope is appropriate for capturing recent patterns of environmental quality, although longer time series may provide additional insight into slower ecological changes and broader climatic oscillations.

Without effective coastal management strategies that incorporate climate change mitigation, vulnerable ecosystems will continue to decline, exacerbating environmental challenges and reducing regional resilience. Santa Catarina has 295 municipalities, 26 of which are coastal. While the State Coastal Management Plan (PEGC/SC) provides a regulatory framework, its implementation varies widely across municipalities (Santa Catarina, 2005; Vieira et al., 2009). In the Tubarão River Basin, urban expansion and tourism-driven development further pressure fragile ecosystems, with inconsistent management leading to ongoing environmental degradation, especially in densely populated areas with weak land-use enforcement (Cristiano et al., 2018; Vieira et al., 2009). While some municipalities successfully integrate coastal management policies, others struggle with limited resources, technical expertise, and conflicting economic interests. These challenges are not unique to Brazil, as effective coastal governance remains a global issue, requiring adaptive policies and stronger enforcement to balance development and conservation (Agrawal et al., 2022). In this sense, RSEI-based assessments provide an operational tool to support adaptive coastal and watershed management under increasing climatic and land-use pressures.

## Conclusion

This study presents an integrated assessment of terrestrial environmental quality in the Tubarão River Basin by combining multi-temporal remote sensing indicators, a PCA-based Remote Sensing Ecological Index (RSEI), spatial clustering, and mixed-effects modeling. The approach allowed the identification of consistent spatial patterns in environmental quality across municipalities and highlighted the role of land-use composition in shaping these patterns. Forest cover was positively associated with higher RSEI values, whereas agricultural and urban land uses exhibited negative effects, with particularly low environmental quality observed in coastal municipalities.

Beyond land-use effects, the results emphasize the importance of spatial heterogeneity, as municipality-level differences explained a substantial portion of the variance in environmental quality. Although land-use patterns remained relatively stable between 2014 and 2023, a generalized increase in land surface temperature was observed across the basin, suggesting that broader climatic pressures may contribute to the observed decline in environmental quality. This reinforces the relevance of integrating thermal indicators into large-scale environmental monitoring frameworks.

By applying a standardized, reproducible methodology based on widely available remote sensing products, this study contributes a robust framework for monitoring environmental quality in coastal river basins. The findings offer valuable insights for territorial planning, environmental management, and policy-making in regions experiencing the combined pressures of land-use change and climate variability. Given the global relevance of these challenges, the methodological approach and results presented here may be adapted to support environmental assessments in other dynamic landscapes worldwide.

## Supplementary Information

Below is the link to the electronic supplementary material.

ESM 1 (XLSX 58.4 KB)

## Data Availability

Data will be made available on reasonable request.
